# Prognostic significance of 5-fluorouracil metabolism-relating enzymes and enhanced chemosensitivity to 5-fluorouracil by 5-chloro 2,4-dihydroxy-pyridine in urothelial carcinoma

**DOI:** 10.1186/1471-2407-12-420

**Published:** 2012-09-22

**Authors:** Hiroki Ide, Eiji Kikuchi, Masanori Hasegawa, Norihide Kozakai, Takeo Kosaka, Akira Miyajima, Mototsugu Oya

**Affiliations:** 1 Department of Urology, Keio University School of Medicine, Tokyo, Japan

**Keywords:** Urothelial carcinoma, S-1, 5-fluorouracil, Thymidylate synthase, Dihydropyrimidine dehydrogenase

## Abstract

**Background:**

Recently, S-1, a novel 5-fluorouracil (5-FU)-based agent containing the strong dihydropyrimidine dehydrogenase (DPD) inhibitor, 5-chloro-2,4-dihydropyrimidine (CDHP) has been clinically used to treat various non-urothelial carcinomas (UC). High levels of thymidylate synthase (TS), the target enzyme of 5-FU and DPD which degrades the majority of 5-FU, are associated with poor prognosis in some cancers. However, only a few reports have dealt with this in UC. The aim of this study was to investigate the clinical significance of TS and DPD in upper tract urothelial carcinoma (UTUC) and evaluate the role of TS and DPD on the sensitivity of 5-FU in UC cell lines and the anti-tumor effect of S-1 in UC xenograft model.

**Methods:**

Firstly, we evaluated the immunohistochemical expression of TS and DPD in 176 patients with UTUC to determine their prognostic significance. Secondly, the levels of TS and DPD in UC cell lines were measured by ELISA and real-time PCR. Furthermore, the association between their levels and the sensitivity to 5-FU was examined using the small interfering RNA (siRNA) specific for TS and DPD. Thirdly, the anti-tumor effect of S-1 was evaluated in UC xenograft model.

**Results:**

Immunohistochemical evaluation of TS and DPD in UTUC human samples revealed that TS expression was significantly associated with stage, grade, and lymphovascular invasion and DPD expression was significantly associated with grade. Multivariate analysis revealed that high TS expression was an independent predictor of disease-specific survival in them. In *in vitro* study using UC cell lines, high levels of TS and DPD were associated with low response to 5-FU and these associations were confirmed with siRNA specific for TS and DPD. In *in vivo* study using UC xenograft model, S-1 treatment dramatically inhibited tumor growth compared to controls, tegafur, or UFT in UC tumor with a high level of DPD.

**Conclusions:**

TS plays an important role in the prognosis of UTUC and S-1 may be a key agent for UC tumor, especially with a high level of DPD.

## Background

Urothelial carcinoma (UC) occurs throughout the urinary tract, including the upper urinary tract, bladder, and urethra, however, most cases of UC involve the bladder. Upper tract urothelial carcinoma (UTUC) is very rare, accounting for approximately 5% of all UC [1]. The prognosis of UC patients with metastasis is poor and 5-year survival rates for bladder cancer with lymph node metastasis and UTUC with it were reported to be 18-29% and 35%, respectively [2,3]. Systemic chemotherapy with a cisplatin-containing regimen is often proposed for patients with metastatic UC. Cisplatin-based chemotherapy has a short-term therapeutic effect against metastatic UC with a response rate of about 50%, however, longer survival after receiving systemic chemotherapy is low, with a 5-year survival rate of only 13-15% [4-6]. Thus, a novel chemotherapeutic regimen for treating highly aggressive UC is urgently needed.

5-Fluorouracil (5-FU), an antitumor pyrimidine, has frequently been used clinically in patients with various cancers, including UC. 5-FU is converted to 5-fluorodeoxyuridine monophosphate and this inhibits thymidylate synthase (TS), which is the enzyme that catalyzes the methylation of deoxyuridine monophosphate to deoxythymodine monophosphate, by forming a stable ternary complex with methylene tetrahydrofolate, and the ternary complex leads to the inhibition of DNA synthesis [7,8]. Several studies in some cancers including bladder cancer have shown that TS activity was greater in cancerous tissue specimens than in normal tissue samples and that the TS activity level was correlated with stage progression [9,10]. Furthermore, it was reported that high TS expression was associated with a poor prognosis in gastric, colon, and bladder cancers [11-13]. However, to our knowledge, there have not been any reports which showed the clinical significance of TS in UTUC.

It was reported that the response-limiting factor for 5-FU was the plasma level of 5-FU. Therefore, continuous infusion was necessary to obtain higher responses because most administered 5-FU was degraded through a catabolic pathway by dihydropyrimidine dehydrogenase (DPD) [14]. Previous studies in some cancers including bladder cancer showed that DPD activity was greater in cancerous tissue specimens than in normal tissue samples and that DPD activity level was correlated with stage progression [15,16]. Furthermore, it was reported that high DPD expression was associated with a poor prognosis in gastric and colon cancers [17,18]. However, no report has ever demonstrated the clinical significance of DPD in UTUC.

Previous reports in gastric and colon cancers showed that high TS expression was significantly related to a low response to 5-FU [19-21]. Other reports in gastric and colon cancer patients found that a high DPD level resulted in a low sensitivity to 5-FU [22-24]. However, to the best of our knowledge, no report has showed an association between TS or DPD level and the sensitivity to 5-FU in UC cells. Recently, DPD-inhibitory fluoropyrimidine (DIF) compounds such as UFT and S-1 have been developed in an attempt to resolve the problem of rapid reduction of 5-FU by DPD. S-1 is a new oral formulation of DIF developed in Japan and consists of a strong DPD inhibitor, 5-chloro-2,4-dihydropyrimidine (CDHP; gimeracil), which is approximately 180 times more potent than the DPD inhibitor uracil, which is a component of UFT. Thus, S-1 results in higher concentrations of 5-FU in the blood and tumor tissue than UFT [25]. According to a previous report, UFT has been used successfully in the study of bladder cancer [26]. Because S-1 is thought to be more potent than UFT with respect to the biochemical modulation effect, one might expect a stronger antitumor effect by using S-1 in UC. However, only a few *in vitro* reports have showed that CDHP enhanced the antitumor activity of 5-FU in bladder cancer cell lines [16,27]. Furthermore, no *in vivo* study has demonstrated the enhancement of antitumor activity of 5-FU by CDHP, i.e., the efficacy of S-1 in UC.

Therefore, in the present study, we evaluated 1) the association between the tumor characteristics of 176 cases of UTUC and the expression of TS and DPD by immunohistochemistry with slides re-reviewed by genitourinary pathologists to determine the clinical role of TS and DPD expression in tumor progression and survival in UTUC. We also examined 2) the level of TS and DPD in UC cell lines and the association between TS or DPD level and the sensitivity to 5-FU *in vitro*. Finally, we evaluated 3) the enhancement of the antitumor activity to 5-FU by CDHP *in vitro* and *in vivo*.

## Methods

### Immunohistochemical evaluation of TS and DPD in UTUC human samples

Surgical specimens from 176 patients who had been surgically treated for UTUC at Keio University Hospital from 1986 to 2007 were examined. The median follow-up was 45 months and the median patient age was 67 years (range, 36–89 years). The patients did not undergo any chemotherapy or radiation therapy prior to the surgery. Patients with distant metastasis at the time of diagnosis and incomplete clinical data were excluded from the study. A nephroureterectomy with the removal of the bladder cuff was the most common procedure (n = 172), while a partial ureterectomy was performed in the remaining 4 patients. Regional lymphadenectomy was generally performed in patients with suspicious lymph nodes on preoperative axial imaging or with adenopathy detected during an intraoperative examination. Extended lymphadenectomy was not routinely performed. The patients were followed postoperatively with urinary cytology every 3 months for 2 years and every 6 months thereafter. Computed tomography as well as cystoscopy and magnetic resonance imaging were performed every 6 months for 5 years and annually thereafter.

Tissue samples were obtained from consented patients in this study which was approved by Keio University Ethics Committee. All specimens were fixed in 10% formalin and embedded in paraffin, and all slides were re-reviewed by genitourinary pathologists. Tumors were staged according to the American Joint Committee on Cancer-Union Internationale Contre le Cancer TNM classification. Tumor grading was assessed according to the 1998 WHO/International Society of Urology Pathology consensus classification [28]. Lymphovascular invasion (LVI) was defined as the presence of tumor cells within an endothelium-lined space without underlying muscular walls.

Sections (4 μm) of formalin-fixed and paraffin-embedded material were analyzed. The sections were deparaffinized in xylene and rehydrated in graded alcohols and distilled water. After antigen retrieval with citric acid (pH 6.0), endogenous peroxidase activity was blocked with 1% hydrogen peroxide for 30 minutes followed by washing with distilled water. To bind nonspecific antigens, the sections were incubated with 5% skim milk for 15 minutes. The sections were incubated with either an anti-TS rabbit monoclonal antibody (1:100 dilution, Taiho Pharmaceutical Co, Tokyo, Japan) at room temperature for 1 hour or an anti-DPD mouse polyclonal antibody (1:100 dilution, Taiho Pharmaceutical Co.) at room temperature for 1 hour. After washing with phosphate-buffered saline (PBS), they were incubated with secondary antibodies against rabbit IgG conjugated to a peroxidase-labeled dextran polymer (no dilution, anti-rabbit Envision, Dako Japan, Tokyo) or against mouse IgG conjugated to a peroxidase-labeled dextran polymer (no dilution, anti-mouse Envision, Dako Japan) for 1 hour. Color was developed with 3,3’-diaminobenzamine tetrahydrochloride in 50 nmol/L Tris–HCl (pH 7.5) containing 0.005% hydrogen peroxidase. The sections were counterstained with hematoxylin.

To evaluate TS and DPD staining, cancer cells with positive staining in the cytoplasm were counted in at least 10 representative fields selected randomly by a uro-pathologist and staining intensity that was stratified from 0 to 3 (0, no staining; 1, weak staining; 2, moderate staining; 3, strong staining) was estimated. Cases with more than 25% positive tumor cells (moderate and strong staining) in a section were regarded as positive expression as previously described [29]. The evaluation of immunostaining was made by a uro-pathologist who was unaware of the clinico-pathological data and clinical outcomes of the patients.

### Cell lines and chemicals

Three human UC cell lines, T24, 5637, and UMUC-3 (American Type Culture Collection, Rockville, MD, USA), were grown in RPMI 1640 supplemented with 10% heat-inactivated fetal bovine serum, 100 μg/ml streptomycin (Life Technologies, Inc., Grand Island, NY, USA), and 100 IU/ml penicillin (Life Technologies, Inc., Grand Island, NY, USA).

5-FU was synthesized by Wako Pure Chemical Industries (Osaka, Japan) and CDHP, tegafur, UFT and S-1 were kindly supplied by Taiho Pharmaceutical Co., Ltd (Tokyo, Japan). 5-FU and CDHP were dissolved in culture medium to prepare a 100 μg/ml solution and subsequently diluted in culture medium. Tegafur, UFT and S-1 were dissolved in distilled water with 0.5% hydroxypropylmethylcellulose (HPMC).

### Cell growth assay

Briefly, 2 × 10^4^ cells were seeded into each well of 96-well plates and allowed to grow overnight. The cells were then treated with various concentrations of 5-FU with or without CDHP. After 72 hours of incubation, cytotoxicity was determined using WST-1; 4-[3-(4-lodophenyl)-2-(4-nitrophenyl)-2 H-5-tetrazolio]-1,3-benzene disulfonate (Takara Bio Inc, Shiga, Japan). The absorbance value of each well was determined at 450 nm with a 650 nm reference beam by a microplate reader (Bio-Rad Laboratories, Inc, Tokyo, Japan). As previously described [25], the 5-FU concentration causing 50% growth inhibition compared with the control (IC_50_) was calculated from the regression line. These experiments were repeatedly performed at different days, where new cells were grown.

### Enzyme-linked immunosorbent assasy (ELISA)

Each sample (1.0 × 10^8^ cells or xenograft tumors) was homogenized in a 10-fold volume of sample weight of the diluting solution (20 mM PBS which contained 0.05% Tween 20) and centrifuged at 105,000× g, 4°C for an hour. The supernatant (100 μl) was then dispensed onto an anti-human TS or DPD polyclonal antibody (Solid phase antibody: Mitsubishi Chemical Medience, Tokyo, Japan) immobilized plate and incubated for an hour at room temperature. After the wells were washed four times with PBS, 100 μl aliquots of horseradish peroxidase were conjugated to anti-human TS or DPD polyclonal antibody (Label antibody: Mitsubishi Chemical Medience, Tokyo, Japan). After the wells were washed four times with PBS, 100 μl aliquots of 0.1 M acetate buffer (pH 5.5; color-developing solution) containing 3 mg/ml orthphenylenediamine and 0.75 mM hydrogen peroxide were added, followed by incubation for 30 minutes in the dark. Finally, 100 μl aliquots of 1 M sulfuric acid were added to terminate the reaction and the measurements were conducted with the measuring wavelength of an ELISA plate reader set at 490 nm.

### Real-time quantitative PCR (RT-PCR)

The cells were lysed with RNAiso reagent (Takara Bio Inc, Shiga, Japan) according to the manufacturer’s directions for total RNA extraction. RNA was quantitated by the ratio of absorbance at 260/280 nm. Reverse transcription of RNA to cDNA was carried out using a High Capacity cDNA Archive Kit (Applied Biosystems, Tokyo, Japan). Next, real time PCR was carried out in a final volume of 20 μl containing cDNA template, TS, DPD or GAPDH primers (Applied Biosystems, Tokyo, Japan) and TaqMan® Universal PCR Master Mix (Applied Biosystems, Tokyo, Japan), and DNase RNAase free water, using a StepOne real time PCR system (Applied Biosystems, Tokyo, Japan) according to the manufacturer’s protocol. Cycling conditions were 50°C for 10 minutes, 95°C for 10 minutes, and then 40 cycles at 95°C for 15 seconds and 60°C for 1 minute. The data were then quantified using the comparative *C*_t_ method for relative gene expression compared with GAPDH as endogenous control. The primers and TaqMan probe sets for TS (TYMS) (Hs00426591_m1), DPD (DPYD) (Hs00559279_m1), and human GAPDH endogenous control (Hs99999905_m1) were purchased from Applied Biosystems.

### Small interfering RNA (siRNA)

Three predesigned siRNAs for DPYD, TYMS and nontargeting control (NTC) siRNA (AllStars Negative Control siRNA) were obtained from Invitrogen Co (Tokyo, Japan). UMUC-3 cells (1.5 × 10^5^ per well) were cultured in antibiotic-free medium overnight at 37°C in 5% CO_2_ and then transfected with each siRNA for DPYD or TYMS or nontargeting control, using Lipofectamine Max (Invitrogen Co, Tokyo, Japan). Forty-eight hours later, the transfected cells were washed and used for subsequent experiments.

### Treatment *in vivo*

All of the procedures involving animals and their care in this study were approved by the Animal Care Committee of Keio University in accordance with institutional and Japanese government guidelines for animal experiments. Female BALB/c-*nu/nu* mice were obtained from Sankyo Lab Service Co. (Tokyo, Japan). UMUC-3 cells (2 × 10^6^) were implanted subcutaneously into the flank of each nude mouse. When a mouse developed a palpable tumor, it was randomly assigned to one of 4 groups. Treatment groups were dosed at the maximal tolerable dose; tegafur 180 mg/kg/day, UFT 20 mg/kg/day, and S-1 8.3 mg/kg/day [25]. Each drug was administered daily by gavage. Control animals only received vehicle by gavage. Each experimental group consisted of 10 mice. The mice were carefully monitored, and tumor size and body weight were measured every 4 days. Tumor volume was calculated according to the formula a^2^ × b × 0.52, where a and b are the smallest and largest diameters, respectively. Eight weeks after tumor cell implantation, the mice were sacrificed and the tumors were collected. The levels of TS and DPD in the tumors were then measured by ELISA as described previously.

### Statistical analysis

The association between TS and DPD, and clinico-pathological features was assessed using χ^2^ test. Disease-specific survival (DSS) and progression-free survival (PFS) were calculated by the Kaplan-Meier method and analyzed by the log-rank test. Cox proportional hazards regression analysis was used to assess the prognostic indicators that included age, gender, tumor location, pT, grade, nodal involvement, LVI, TS expression, and DPD expression for survival. The difference between two groups in *in vitro* study and in the animal model was assessed with the Mann–Whitney *U*-test. The level of statistical significance was set at P <0.05. These analyses were performed with an SPSS Version 16.0 statistical software package (SPSS Corporation).

## Results

### The clinical role of TS and DPD expression in UTUC human samples

#### TS and DPD expression in UTUC

**Figure 1:**
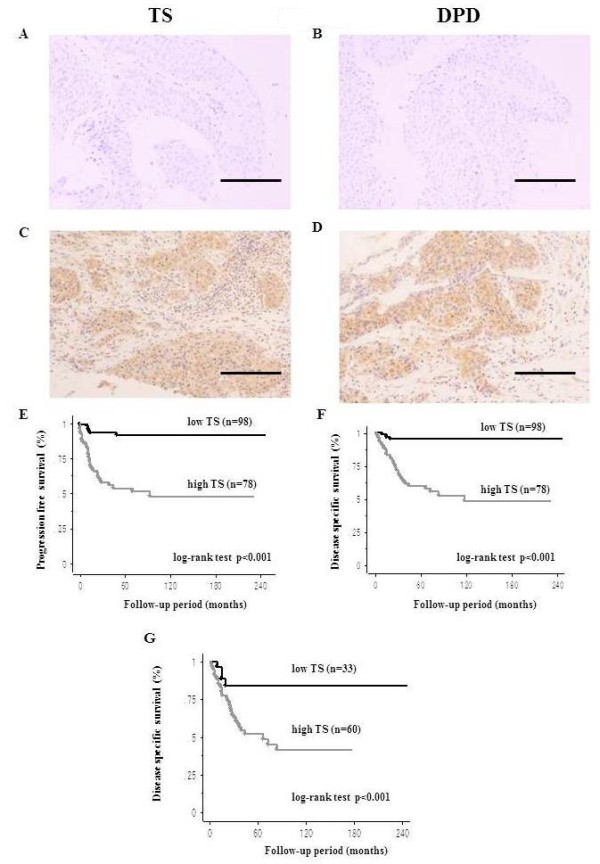
**Immunostaining for TS and DPD in UTUC.** Immunostaining for TS in pTa and low grade UTUC (**A**), and pT3 and high grade UTUC (**C**). Immunostaining for DPD in pTa and low grade UTUC (**B**), and pT3 and high grade UTUC (**D**). Bar indicates 0.1 mm. Kaplan-Meier analysis of progression-free survival (**E**) and disease-specific survival (**F**) of the overall patients treated with surgery for UTUC according to TS expression. Kaplan-Meier curves of the disease-specific survival (**G**) of the patients with pathological stage T2 or higher according to TS expression.

All the specimens were fixed in 10% formalin and embedded in paraffin, and all slides were re-reviewed by genitourinary pathologists. To elucidate the clinical significance of TS and DPD in UTUC, we examined the immunohistochemical expression of TS and DPD (Figure 1A-D). TS staining was weak in pTa and low grade UTUC (Figure 1A). On the other hand, in the pT3 and high grade UTUC, strong TS staining was observed in many of the cancer cells (Figure 1C). DPD staining was weak in the low grade UTUC (Figure 1B). On the other hand, in the high grade UTUC, strong DPD staining was observed in many of the cancer cells (Figure 1D).

#### Association between TS and DPD expression and clinico-pathological features

We analyzed the association between TS and DPD expression and the clinicopathological features of 176 UTUC samples (Table 1). The TS expression levels were significantly associated with pT-stage (p < 0.001), grade (p < 0.001), LVI (p < 0.001), and lymph node metastasis (p < 0.001). DPD expression was significantly associated with tumor grade (p < 0.001).

**Table 1:** Association of clinicopathological parameters and TS or DPD expression

**Parameter**	**TS**		**P value**	**DPD**		**P value**
	**Negative, n (%)**	**Positive, n (%)**		**Negative, n (%)**	**Positive, n (%)**	
Age						
< 65 years (n = 75)	38(38.8)	37(47.4)	0.248	48(46.6)	27(37.0)	0.219
≥65 years (n = 101)	60(61.2)	41(52.6)		55(53.4)	46(63.0)	
Gender						
Male (n = 132)	72(73.5)	60(76.9)	0.599	75(72.8)	57(78.1)	0.482
Female (n = 44)	26(26.5)	18(23.1)		28(27.2)	16(21.9)	
Pathological T stage						
pTa	35	8	<0.001	33	10	0.111
pT1	30	10		20	20	
pT2	19	10		18	11	
pT3	13	46		30	29	
pT4	1	4		2	3	
Grade						
G1	0	0	<0.001	0	0	<0.001
G2	60	19		59	20	
G3	38	59		44	53	
LVI						
Negative (n = 108)	82(83.7)	26(33.3)	<0.001	68(66.0)	40(54.8)	0.158
Positive (n = 68)	16(16.3)	52(66.7)		35(34.0)	33(45.2)	
Nodal involvement						
Negative (n = 157)	94(95.9)	63(80.8)	<0.001	94(91.3)	63(86.3)	0.102
Positive (n = 19)	4(4.1)	15(19.2)		9(8.7)	10(13.7)	
Tumor location						
Pelvis (n = 112)	56(57.1)	56(71.8)	0.058	73(70.9)	39(53.4)	0.062
Ureter (n = 64)	42(42.9)	22(28.2)		30(29.1)	34(46.6)	

TS: thymidylate synthase; DPD: dihydropyrimidine dehydrogenase; LVI: lymphovascular invasion.

#### Prognostic significance of TS expression in overall UTUC patients

To examine the prognostic value of TS and DPD expression in UTUC patients, univariate and multivariate analyses were performed ( Additional file 1). Univariate analysis revealed that the pT-stage, grade, LVI, lymph node metastasis, and TS were all significant predictors of the PFS and DSS. Multivariate Cox regression analysis showed that pT-stage, LVI, and TS expression were independent prognostic indicators of PFS and DSS. Kaplan-Meier curves also demonstrated significant differences in PFS (Figure 1E, p < 0.001) and DSS (Figure 1F, p < 0.001) in regard to TS expression. The 5-year PFS and DSS rates were 53.8% and 60.0%, respectively, for patients with higher TS expression, compared with 91.4% and 95.1% for patients with lower TS expression. Kaplan-Meier analysis demonstrated no significant differences between higher and lower levels of DPD for PFS (p = 0.316) and DSS (p = 0.165) (data not shown).

#### Prognostic significance of TS expression in UTUC patients with pathological stage T2 or higher

In a subgroup of patients with pathological stage T2 or higher (n = 93), multivariate analysis demonstrated that LVI and TS expression were independently associated with PFS and DSS. The 5-year DSS rates in patients with high TS expression were 51.5%, compared with 83.9% in patients with low TS expression (p <0.001; Figure 1G).

### The in vitro study using UC cell lines

#### The levels of TS and DPD in UC cell lines

The levels of TS and DPD in three UC cell lines are shown in Additional file 2. The median TS protein level in T24 cells was 2375 (range:2283–2475) ng/mg, which was the highest among the three cell lines. The relative TS mRNA levels exhibited the same pattern as the protein levels. The median DPD protein and relative DPD mRNA levels in UMUC-3 cells (232.6 ng/mg protein, 13.0; DPD/GAPDH ratio) were the highest among the three cell lines.

#### Cytotoxic effects of 5-FU treatment with or without CDHP

**Figure 2:**
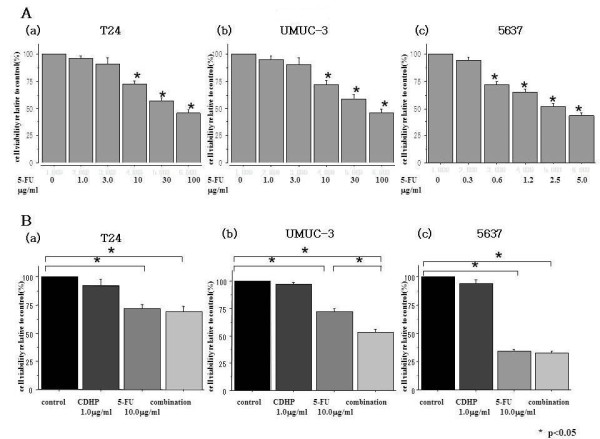
**Cytotoxic effects of 5-FU with or without CDHP on UC cell lines. **(**A**) Cytotoxic effects of various concentrations of 5-FU and (**B**) 5-FU (10.0 μg/ml) in combination with CDHP (1μg/ml) on 3 bladder cancer cell lines (**a**; T24, **b**; UMUC-3 and **c**; 5637). Cell viability was measured by WST assay. Each value represents the mean derived from at least three individual experiments; bars ± S.E.*, more statistically significant than controls (p < 0.05).

In T24 and UMUC-3 cells, 5-FU at concentrations of 10 μg/ml or higher had significant cytotoxic effects (Figure 2Aa, b). Meanwhile, in 5637 cells, significant growth inhibition by 5-FU was seen at concentrations of 0.6 μg/ml or higher (Figure 2Ac). CDHP alone did not demonstrate a cytotoxic effect in bladder cancer cells tested at concentrations of 30 μg/ml or lower (data not shown).

After 72 hours of concurrent treatment of 5-FU and CDHP, cytotoxicity was determined in T24, UMUC-3 and 5637 cells. In UMUC-3 cells, the combination treatment of 5-FU (10 μg/ml) with CDHP (1 μg/ml) resulted in significantly higher levels (45.6%) of cell growth inhibition compared to the treatment with 5-FU (10 μg/ml) alone (26.9%) (p < 0.05; Figure 2Bb).

Meanwhile, there were no significant differences in cell growth inhibition between the combination treatment of 5-FU (10 μg/ml) with CDHP (1 μg/ml) (32.6% and 67.2%) and the treatment with 5-FU (10 μg/ml) alone (25.6% and 65.5%) in T24 and 5637 cells, respectively (Figure 2Ba,c).

Additional file 2 shows that the values of IC_50_ for 5-FU with or without CDHP (1 μg/ml) in the three cell lines. It was also observed that the value of IC_50_ of 5-FU in UMUC-3 cells treated with 5-FU and CDHP (25.0 ± 0.8 μg/ml) was significantly lower than that in UMUC-3 cells treated with 5-FU alone (53.4 ± 1.4 μg/ml) (p < 0.05). Meanwhile, there were no significant differences in the IC_50_ between the combination treatment of 5-FU with CDHP (54.5 ± 2.1 and 2.8 ± 0.3 μg/ml) and treatment with 5-FU alone (58.3 ± 1.8 and 3.0 ± 0.3 μg/ml) in T24 and 5637 cells, respectively. The addition of 1 μg/ml of CDHP enhanced the growth inhibitory effect of 5-FU by 2.1-fold in UMUC-3 cells. Also, no significant cytotoxic enhancement by CDHP was observed in 5-FU treatment in T24 and 5637 cells.

#### Effects of siRNA for DPD and TS on the sensitivity to 5-FU in UMUC-3 cells

**Figure 3:**
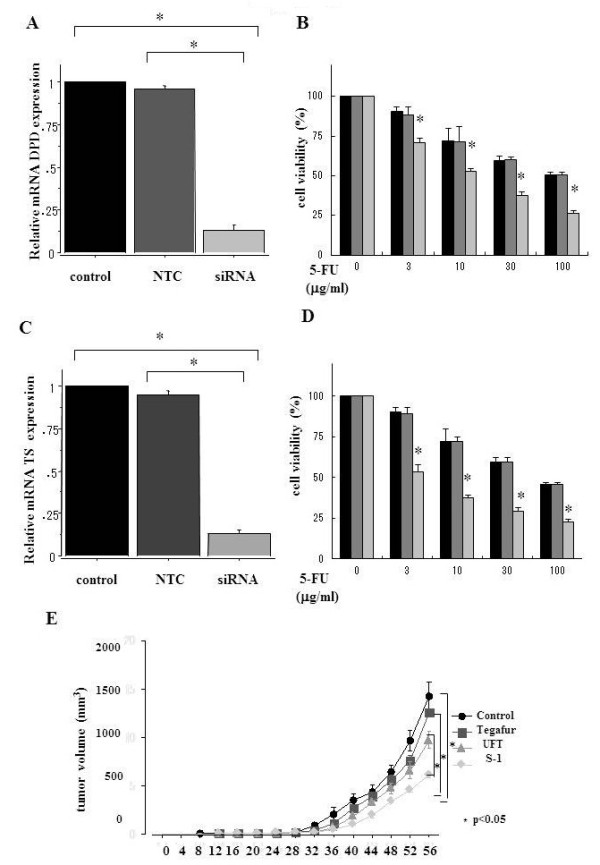
**Effects of siRNA specific for DPD or TS on sensitivity of UC cells to 5-FU and the antitumor effect of S-1 in UC xenograft model. **(**A**) Expression of DPD mRNA in UMUC-3 cells treated with or without siRNA targeting DPD. Nontargeting control (NTC) siRNA was also used as a negative control. (**B**) Effects of DPD siRNA on sensitivity of UMUC-3 cells to 5-FU. UMUC-3 cells transfected with siRNA targeting DPD or NTC were treated with various concentrations of 5-FU. The cell viabilities were measured by WST assay. (**C**) Expression of TS mRNA in UMUC-3 cells treated with or without siRNA targeting TS. (**D**) Effects of TS siRNA on sensitivity of UMUC-3 cells to 5-FU. UMUC-3 cells transfected with siRNA targeting TS or NTC were treated with various concentrations of 5-FU. A-D, Each value represents the mean derived from at least three individual experiments *, more statistically significant than controls (p < 0.05); bars ± SE. (**E**) Treatment *in vivo*. UMUC-3 cells (2 × 10^6^) were implanted s.c. into the flank of nude mice. When a palpable tumor had developed, S-1 (8.3 mg/kg), UFT (20 mg/kg) and tegafur (180 mg/kg) were administered orally once daily. Control animals were administered vehicle alone. Mean tumor volumes in mm^3^ are shown. E, *, more statistically significant than UFT, tegafur and controls (p < 0.05); bars ± SE.

Transfection of siRNA for DPD reduced the mRNA level of DPD to 10% in UMUC-3 cells (p < 0.05) whereas no significant difference was observed by transfection with NTC compared to vehicle control (Figure 3A). 5-FU (3 μg/ml or higher) treatment in UMUC-3 cells transfected with siRNA for DPD resulted in significantly higher levels of cell growth inhibition compared to that in UMUC-3 cells transfected with NTC or treated with vehicle control (Figure 3B). Also, the IC_50_ value for 5-FU in UMUC-3 cells transfected with siRNA for DPD (9.6 ± 0.4 μg/ml) was significantly lower than that treated with vehicle control (53.4 ± 1.4 μg/ml) or transfected with NTC (52.1 ± 1.2 μg/ml).

Transfection of siRNA for TS reduced the mRNA level of DPD to 10% in UMUC-3 cells (p < 0.05) whereas no significant difference was observed by transfection with NTC compared to control (Figure 3C). 5-FU (3 μg/ml or higher) treatment in UMUC-3 cells transfected with siRNA for TS resulted in significantly higher levels of cell growth inhibition compared to that in UMUC-3 cells transfected with NTC or treated with vehicle control (Figure 3D). Furthermore, the IC_50_ value for 5-FU in UMUC-3 cells transfected with siRNA for TS (4.5 ± 0.5 μg/ml) was significantly lower than that treated with vehicle control (53.6 ± 1.2 μg/ml) or transfected with NTC (52.3 ± 1.0 μg/ml).

### The in vivo study using UC xenograft model

*In vivo* experiments on UMUC-3 tumors showed significant decreases in mean tumor volume treated with S-1 (547 ± 79 mm^3^), compared to controls treated with medium vehicle alone (1525 ± 329 mm^3^), tegafur (1438 ± 337 mm^3^), or UFT (1072 ± 191 mm^3^) 56 days post tumor implantation (*p <* 0.05 for all) (Figure 3E).

The levels of TS and DPD protein in xenograft tumors were shown in Table 2. The median TS level in S-1 (511 : range 487–531 ng/mg protein) was significantly higher than that in control (395 : range 386–425 ng/mg protein), tegafur (412 : range 402–423 ng/mg protein) or UFT (435 : range 411–455 ng/mg protein) (*p <* 0.05 for all). On the other hand, the median DPD level (351 : range 331–382 ng/mg protein) in S-1 was significantly lower than that in control (532 : range 511–552 ng/mg protein), tegafur (495 : range 474–535 ng/mg protein) or UFT (454 : range 431–472 ng/mg protein). (*p <* 0.05 for all).

**Table 2:** Median and inter-quartile range of levels of TS and DPD protein in xenograft tumors

	**TS (ng/mg protein)**	**DPD (ng/mg protein)**
Control	395 (386–425)	532 (511–552)
Tegafur	412 (402–423)	495 (474–535)
UFT	435 (412–455)	454*,** (431–472)
S-1	511*,**,*** (487–531)	351*,**,*** (331–382)

*p < 0.05 compared with control; **p < 0.05 compared with tegafur. ***p < 0.05 compared with UFT.

TS: thymidylate synthase; DPD: dihydropyrimidine dehydrogenase.

## Discussion

### The clinical role of TS and DPD expression in UTUC human samples

Recently, some reports about the association between the level of TS and DPD and prognosis in some cancers including UC have ever been found [11-13,16-18,29]. In our present study, we retrospectively analyzed the impact of TS and DPD protein expression by immunohistochemistry especially in patients with UTUC. The results indicated that TS expression was closely related to tumor grade, stage, and LVI. On the other hand, DPD expression was closely related to tumor grade. To our knowledge, this is the first study examining the expression pattern of TS and DPD in case-matched large UTUC specimens. Recently, histologic evidence of TS expression in human tissue samples has also been reported in various cancers and it has been reported that TS expression was a significant prognostic indicator in gastric and colon cancers [11,12]. Additionally, it has also been reported that the level of TS is correlated with both progression of the stage and increase in the grade, and that TS expression was a significant prognostic indicator of recurrence free survival in patients with bladder cancer [13,29]. In this study, we demonstrated that higher TS expression was significantly associated with a lower PFS and DSS and was an independent prognostic factor in UTUC. These results indicate that the role of TS in UTUC might be of special importance for its progression and the establishment of metastasis.

On the other hand, histologic evidence for DPD expression in human tissue samples has also been reported in various cancers and it has been reported that DPD expression was a significant prognostic indicator in breast and colon cancers [17,18]. Additionally, it has also been reported that the level of DPD was correlated with both progression of the stage and increase in the grade of bladder cancer. However, according to previous reports, DPD expression was not a significant prognostic indicator of recurrence free survival in patients with bladder cancer [16]. In the present study, we found a significant association between DPD expression and grade in UTUC. However, we showed that DPD expression was not significantly associated with PFS and DSS in UTUC. This result was almost the same as that for bladder cancer in a previous report [16].

### The in vitro study using UC cell lines

There is some evidence in the literature indicating that an association between the levels of TS and DPD and the sensitivity to 5-FU. Previous reports found that the TS and DPD levels were significantly correlated with 5-FU sensitivity, with high TS and DPD levels resulting in low sensitivity to 5-FU in various cancers [19-24]. Our study showed that the TS level was the highest in T24 cells and the DPD level was the highest in UMUC-3 cells. Furthermore, the sensitivity to 5-FU in T24 and UMUC-3 cells was lower than that in 5637 cells. Therefore, the high TS and DPD levels in the present study may have resulted in low sensitivity to 5-FU in UC cells. In the present study, targeting of TS and DPD mRNA expression in UMUC-3 cells with siRNA down-regulated the mRNA expression and enhanced sensitivity to 5-FU using WST-assay. Thus, from these results, we demonstrated that there was a close correlation between the levels of TS and DPD and the sensitivity to 5-FU in UC cells.

### The in vivo study using UC xenograft model

Recently, it was reported that S-1 containing CDHP had a significantly higher anti-tumor effect than UFT in various cancers [25]. In fact, S-1 has been clinically used in various cancers, such as of the stomach, colon, lung and pancreas. However, in UC, S-1 is not clinically used at present. Our *in vitro* study demonstrated the enhancement of sensitivity to 5-FU by CDHP in UMUC-3 cells. Furthermore, in *in vivo* study, S-1 had more anti-tumor effect than UFT. Thus, these results indicate that S-1 is more effective than UFT, particularly in UC tumor with a higher DPD level.

With respect to the levels of TS and DPD in tumors treated with 5-FU, it was reported that the TS level was significantly higher and the DPD level was significantly lower than those in control in patients with colorectal cancer [30]. Our study also showed that the level of TS protein in tumors treated with S-1 was significantly higher than that in tumors treated with vehicle alone, tegafur or UFT. On the other hand, the level of DPD in tumors treated with S-1 was significantly lower than that in tumors treated with vehicle alone, tegafur or UFT. Thus, the result of our present study exhibited almost the same pattern as that of the previous study in patients with colorectal cancer.

## Conclusions

In summary, in the present study, it was shown that TS expression was an independent predictor of progression and survival in patients with UTUC. Moreover, using siRNA specific for TS/DPD, a strong relationship between the levels of TS and DPD, and the sensitivity of UC cells to 5-FU was observed. Thus, the measurement of TS and DPD in tumor tissue might raise the possibility of achieving tailor-made medicine with 5-FU-related medicines in UC. Furthermore, S-1 including a strong DPD inhibitor, had a significant inhibitory effect against the growth of UC xenograft tumors with higher DPD levels. This suggests that S-1 might be an alternative therapeutic modality for UC, for which cisplatin-based chemotherapy is the only effective regimen, especially in tumors with a high DPD level.

## Competing interests

The authors declare that they have no competing interests.

## Authors’ contributions

HI was responsible for study design, experimental job, interpretation of the results and writing the manuscript. EK contributed towards the conception and design of the study, interpretation of the results and critically reviewed and edited the manuscript. MH was responsible for data collection. NH, TK, AM and MO were responsible for data analysis and interpretation of the study. All authors read and approved the final manuscript.

## Pre-publication history

The pre-publication history for this paper can be accessed here:

http://www.biomedcentral.com/1471-2407/12/420/prepub

## Supplementary Material

Additional file 1 **Cox regression analysis of progression-free anddisease-specific survival.** LVI: lymphovascular invasion; TS: thymidylate synthase; DPD: dihydropyrimidine dehydrogenase; HR: hazard ratio; CI: confidence interval. Click here for file

Additional file 2 **Median and inter-quartile range of levels of TS and DPD and effect of CDHP on 5-FU cytotoxicity in UC cell lines.** TS: thymidylate synthase; DPD: dihydropyrimidine dehydrogenase; GAPDH: glyceraldehyde-3-phosphate dehydrogenase; CDHP: 5-chloro-2,4-dihydroxypyridine. Click here for file
