# Supplementary material for: Prognostic significance of 5-fluorouracil metabolism-relating enzymes and enhanced chemosensitivity to 5-fluorouracil by 5-chloro 2,4-dihydroxy-pyridine in urothelial carcinoma
Source: BMC Cancer. 2012 Sep 22;12:420. doi: 10.1186/1471-2407-12-420 (PMC3522564; doi:10.1186/1471-2407-12-420)
Supplement: Additional file 1 — Cox regression analysis of progression-free anddisease-specific survival. LVI: lymphovascular invasion; TS: thymidylate synthase; DPD: dihydropyrimidine dehydrogenase; HR: hazard ratio; CI: confidence interval. [file 1471-2407-12-420-S1.doc]

**Additional file 1** Cox regression analysis of progression-free and disease-specific survival

| Parameter | Progression-free survival | | | | | Disease-specific survival | | | | |
| --- | --- | --- | --- | --- | --- | --- | --- | --- | --- | --- |
|  | Univariate | Multivariate | | | | Univariate | Multivariate | | | |
|  | *P* | Parameter Estimate | HR | 95% CI | *P* | *P* | Parameter Estimate | HR | 95% CI | *P* |
| Age  (<65 vs.≧65) | 0.942 |  |  |  |  | 0.437 |  |  |  |  |
| Gender （male vs. female） | 0.482 |  |  |  |  | 0.560 |  |  |  |  |
| Pathological T stage  （≦T2 vs. >T2） | <0.001 | 0.852 | 2.344 | 1.037-  5.303 | 0.041 | <0.001 | 1.042 | 2.834 | 1.152-  6.976 | 0.023 |
| Grade  (G1-G2 vs. G3) | <0.001 |  |  |  |  | <0.001 |  |  |  |  |
| LVI  (negative vs. positive) | <0.001 | 1.140 | 3.126 | 1.306-  7.478 | 0.010 | <0.001 | 1.102 | 3.010 | 1.164-  7.783 | 0.023 |
| TS  (negative vs. positive) | <0.001 | 1.040 | 2.828 | 1.016-  7.875 | 0.047 | <0.001 | 1.207 | 3.342 | 1.006-  11.105 | 0.049 |
| DPD  (negative vs. positive) | 0.768 |  |  |  |  | 0.813 |  |  |  |  |
| Nodal involvement  (presence or absence) | <0.001 |  |  |  |  | <0.001 |  |  |  |  |
| Tumor location  (pelvis or ureter) | 0.231 |  |  |  |  | 0.349 |  |  |  |  |

LVI: lymphovascular invasion; TS: thymidylate synthase; DPD: dihydropyrimidine dehydrogenase; HR: hazard ratio; CI: confidence interval
