# Supplementary material for: Prognostic significance of 5-fluorouracil metabolism-relating enzymes and enhanced chemosensitivity to 5-fluorouracil by 5-chloro 2,4-dihydroxy-pyridine in urothelial carcinoma
Source: BMC Cancer. 2012 Sep 22;12:420. doi: 10.1186/1471-2407-12-420 (PMC3522564; doi:10.1186/1471-2407-12-420)
Supplement: Additional file 2 — Median and inter-quartile range of levels of TS and DPD and effect of CDHP on 5-FU cytotoxicity in UC cell lines. TS: thymidylate synthase; DPD: dihydropyrimidine dehydrogenase; GAPDH: glyceraldehyde-3-phosphate dehydrogenase; CDHP: 5-chloro-2,4-dihydroxypyridine. [file 1471-2407-12-420-S2.doc]

**Additional file 2** Median and inter-quartile range of levels of TS and DPD and effect of CDHP on 5-FU cytotoxicity in UC cell lines

|  | TS | | DPD | | IC_50_ of 5-FU(μg/ml) | | | Enhancement factor |
| --- | --- | --- | --- | --- | --- | --- | --- | --- |
|  | protein  (ng/mg protein) | mRNA  (TS/GAPDH  RT-PCR product ratio) | protein  (ng/mg protein) | mRNA  (DPD/GAPDH　RT-PCR　product ratio) | -CDHP | +CDHP  (1μg/ml) | p value |  |
| T24 | 2375  (2283-2475) | 340.8  (320.1-361.2) | 27.0  (23.1-30.2) | 0.2  (0.1-0.3) | 58.3±1.8 | 54.5±2.1 | 0.101 | 1.1±0.1 |
| UMUC-3 | 690.3  (632.1-750.5) | 13.0  (12.5-13.7) | 232.6  (220.5-248.1) | 13.0  (12.8-13.4) | 53.4±1.4 | 25.0±0.8 | 0.007 | 2.1±0.2 |
| 5637 | 605.1  (552.2-651.5) | 11.0  (10.5-11.3) | 10.1  (7.0-13.3) | 0.1  (0.1-0.2) | 3.0±0.3 | 2.8±0.3 | 0.300 | 1.1±0.1 |

TS: thymidylate synthase; DPD: dihydropyrimidine dehydrogenase; GAPDH: glyceraldehyde-3-phosphate dehydrogenase; CDHP: 5-chloro-2,4-dihydroxypyridine.
